# Long-term outcomes of switching to fixed-dose abacavir/lamivudine (ABC/3TC) or tenofovir/emtricitabine (TDF/FTC): 3-year results of the BICOMBO study

**DOI:** 10.1186/1758-2652-13-S4-P43

**Published:** 2010-11-08

**Authors:** E Martínez, JA Arranz, D Podzamczer, M Lonca, J Sanz, P Barragán, H Knobel, E Ribera, F Gutierrez, S Valero, B Clotet, D Dalmau, F Segura, JR Arribas, P Barrufet, I Santos, A Payeras, E de Lazzari, J Pich, J Gatell

**Affiliations:** 1 Hospital Clínic-IDIBAPS, Barcelona, Spain; 2 Hospital Principe de Asturias, Alcala de Henares, Spain; 3 Hospital de Bellvitge, L'Hospitalet de Llobregat, Spain; 4 Hospital del Mar, Barcelona, Spain; 5 Hospital de Vall d'Hebron, Barcelona, Spain; 6 Hospital Universitario de Elche, Elche, Spain; 7 Hospital Sant Jaume, Calella, Spain; 8 Hospital Germans Trias i Pujol, Badalona, Spain; 9 Hospital de Mutua de Terrassa, Terrassa, Spain; 10 Hospital Parc Tauli, Sabadell, Spain; 11 Hospital La Paz, Madrid, Spain; 12 Hospital de Mataro, Mataro, Spain; 13 Hospital Universitario de La Princesa, Madrid, Spain; 14 Hospital Son Llatzer, Palma de Mallorca, Spain

## Background

Once-daily fixed-dose combinations ABC/3TC and TDF/FTC are the preferred backbones in Europe [1]. Long-term (>2 years) efficacy and safety of these compounds in simplification strategies are unknown.

## Methods

333 HIV-1-infected adults on 3TC-containing triple regimens with <200 copies/mL for at least 6 months had their NRTI backbone randomly switched to either ABC/3TC or TDF/FTC. Pre-planned results at 1 year have been already published [2]. Treatment failure (defined as virological failure, discontinuation of study therapy, withdrawal of consent, lost to follow-up, progression to AIDS, or death), virological failure (defined as confirmed plasma HIV-1 RNA >200 copies/mL), adverse events, and changes in CD4 cells, fasting plasma lipids, glomerular filtration rate (GFR) (Cockcroft-Gault), and transaminases at 3 years were compared between arms.

## Results

Treatment failure increased from 32 (19%) to 58 (35%) patients on ABC/3TC and from 22 (13%) to 61 (37%) patients on TDF/FTC at 1 and 3 years, respectively (HR for ABC/3TC treatment failure at 3 years 0.99, 95% CI 0.69-1.41). The most common reasons for treatment failure at 3 years in both arms were lost to follow-up/withdrawal of consent (68 patients, 20%) or discontinuation of study drugs for reasons other than adverse events (15 patients, 5%). Total discontinuations due to adverse events increased from 17 at 1-year to 18 patients at 3-years on ABC/3TC and from 9 at 1-year to 10 patients at 3-years on TDF/FTC. Total virological failures increased from 4 at 1-year to 6 patients at 3-years on ABC/3TC while no patient on TDF/FTC developed virological failure at 1-year and through 3-years (HR for ABC/3TC virological failure at 3 years 3.59, 95% CI 0.77-6.42). Change from baseline (mg/dL) in triglycerides (+1 vs -29, P=0.008), total cholesterol (+12 vs -12, P<0.001), LDL-cholesterol (+1 vs -1, P<0.001), and HDL-cholesterol (+3 vs -2, P<0.001) were increased in patients on ABC/3TC compared with decreases in patients on TDF/FTC, although total-to-HDL cholesterol ratio remained almost identical in both arms. There were no significant changes in GFR or transaminases in each arm at 3-years.

## Conclusion

From the 1-year analysis, we observed two additional virological failures in patients on ABC/3TC; there were no virological failures in patients on TDF/FTC over 3 years. Through 3 years long-term safety/tolerability was very good. Differential lipid effects between arms were maintained at 3 years.
